# A Novel Serum Glycobiomarker for Diagnosis and Prognosis of Cholangiocarcinoma Detected by *Butea monosperma* Agglutinin

**DOI:** 10.3390/molecules26092782

**Published:** 2021-05-08

**Authors:** Karuntarat Teeravirote, Sukanya Luang, Sakda Waraasawapati, Patcharee Boonsiri, Chaisiri Wongkham, Sopit Wongkham, Atit Silsirivanit

**Affiliations:** 1 Department of Biochemistry, Faculty of Medicine, Khon Kaen University, Khon Kaen 40002, Thailand; teeravirotek@gmail.com (K.T.); sukany@kku.ac.th (S.L.); patcha_b@kku.ac.th (P.B.); chaisiri@kku.ac.th (C.W.); sopit@kku.ac.th (S.W.); 2 Cholangiocarcinoma Research Institute, Khon Kaen University, Khon Kaen 40002, Thailand; sakdawa@kku.ac.th; 3 Department of Pathology, Faculty of Medicine, Khon Kaen University, Khon Kaen 40002, Thailand; 4 Center for Translational Medicine, Faculty of Medicine, Khon Kaen University, Khon Kaen 40002, Thailand

**Keywords:** *Butea monosperma*, cholangiocarcinoma, glycosylation, glycan, tumor marker, lectin

## Abstract

Plant lectins are widely used in medical glycosciences and glycotechnology. Many lectin-based techniques have been applied for the detection of disease-associated glycans and glycoconjugates. In this study, *Butea monosperma* agglutinin (BMA), a lectin purified from seeds of the medicinal plant *Butea monosperma*, was used for the detection of cholangiocarcinoma (CCA)-associated glycans. Expression of BMA-binding *N*-acetyl galactosamine/galactose (GalNAc/Gal)-associated glycan (BMAG) in CCA tissues was determined using BMA lectin histochemistry; the results showed that BMAG was undetectable in normal bile ducts and drastically increased in preneoplastic bile ducts and CCA. The study in hamsters showed that an increase of BMAG was associated with carcinogenesis of CCA. Using an in-house double BMA sandwich enzyme-linked lectin assay, BMAG was highly detected in the sera of CCA patients. The level of serum BMAG in CCA patients (N = 83) was significantly higher than non-CCA controls (N = 287) and it was applicable for diagnosis of CCA with 55.4% sensitivity, 81.9% specificity, and 76.0% accuracy. A high level of serum BMAG (≥82.5 AU/mL) was associated with unfavorable survival of CCA patients; this information suggested the potential of serum BMAG as a poor prognostic indicator of CCA. In summary, BMAG was aberrantly expressed in preneoplastic bile ducts and CCA, it was also highly detected in patient serum which potentially used as a marker for diagnosis and prognostic prediction of CCA.

## 1. Introduction

Cholangiocarcinoma (CCA), a cancer arising from bile duct epithelium, is one of the most common primary liver cancers in North-Eastern Thailand [1]. Early detection of CCA is still a challenging task for CCA management due to the non-specific signs and symptoms and the lack of a sensitive/specific marker for detection of the disease. Tumor-associated glycans are expressed as a consequence of aberrant glycosylation and have been used as the biomarkers for detection of cancer. Glycomic analyses of CCA cell lines and patient tissues using lectin-based approaches indicated the occurrence of aberrant glycosylation in CCA [2,3]. Several CCA-associated glycans such as carbohydrate antigen 19-9 (CA19-9), CA-S121, CA-S27 and carcinoembryonic antigen have been used as the glycobiomarkers or candidates for diagnosis, monitoring and prognostic prediction of CCA [4,5,6].

Based on carbohydrate binding properties, lectins are widely used for glycobiology research in several aspects, e.g., functional analysis of specific glycans and glycoproteins, affinity purification of glycans and detection of disease-associated glycobiomarkers. Many plant lectins, for instance sialic acid-binding lectins (e.g., *Maackia amurensis* lectin-II and *Sambucus nigra* agglutinin) and *N*-acetyl galactosamine/galactose (GalNAc/Gal) binding lectins (e.g., soybean agglutinin, *Sophora japonica* agglutinin (SJA), *Vicia villosa* lectin, and *Wisteria floribunda* agglutinin), have been used to detect CCA-associated glycans and glycoproteins in tumor tissues and sera from CCA patients [2,5,6,7,8,9,10]. Considering the reactivity of these lectins in CCA, GalNAc/Gal-associated glycans could be a potential biomarker for CCA diagnosis and prognostic prediction. They were highly detected in patient sera, although the level of these glycans in the sera of CCA patients were variable.

In this study, *Butea monosperma* agglutinin (BMA), a GalNAc/Gal binding lectin from *Butea monosperma* [11,12], was used to detect the expression of CCA-associated glycans in patients’ tissues and sera. A double BMA sandwich enzyme-linked lectin assay was developed to measure BMA-binding glycan (BMAG) levels in the sera. The potential of using serum BMAG level as a diagnostic and prognostic marker of CCA was investigated.

## 2. Results

### 2.1. BMAG Was Significantly Increased in Pre-Neoplastic Bile Ducts and CCA Cells

**Figure 1:**
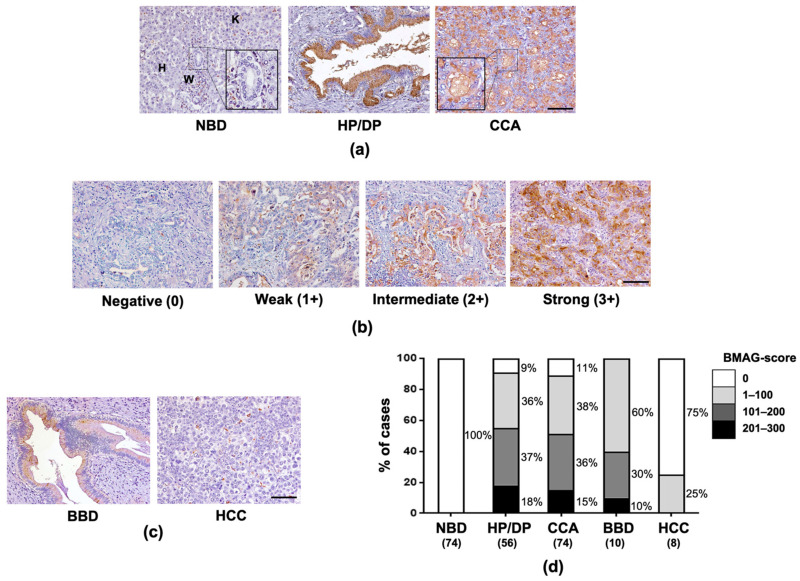
Expression of BMAG in patient tissues. (**a**) BMA histochemistry of CCA tissues demonstrated the expression of BMAG in normal bile ducts (NBD, inlet figure), hepatocytes (H), inflammatory cells (W), Kupffer cells (K) and hyperplastic/dysplastic (HP/DP) bile ducts and CCA. (**b**) BMAG staining in CCA was varied from negative (0), weakly positive (1+), moderately positive (2+), strongly positive (3+). (**c**) BMAG expression was determined in tissues from benign biliary diseases (BBD) and hepatoma (HCC). (**d**) Bar-graph representing the percentage of cases with different BMAG-score, total case number in each group was showed in parentheses. The scale-bar (**—**) equals 100 µm.

BMAG expression in CCA patients’ tissues was determined using BMA histochemistry. BMAG signal was undetectable in normal bile ducts, hepatocytes and inflammatory cells, while it was highly detected in hyperplastic/dysplastic (HP/DP) bile ducts and CCA cells (Figure 1a). In CCA tissues, the positive signal of BMAG was observed mainly in the cytoplasm of CCA cells (Figure 1a,b). In some cases, BMAG signal was also detected in the luminal substances (Figure 1a, inlet), suggesting the possibility of detecting BMAG in patient serum and bile.

Patients’ CCA tissues displayed different levels of BMAG expression, from negative to strong positive staining (Figure 1b). The positive signal of BMAG was found in 91.1% (51/56) of HP/DP bile ducts and 89.2% (66/74) of tumors in CCA tissues. BMAG expression was also found in HP/DP bile ducts from benign biliary diseases (BBD) (Figure 1c). In contrast to BBD and CCA, hepatoma rarely expressed BMAG, as the positive staining for BMAG was seen only in 25% (2/8) of the hepatoma cases (Figure 1c,d). To analyze the correlation between tissue BMAG expression and clinicopathological data, CCA patients were classified into (<107) and high (≥107) according to the median of tissue BMAG score. Using χ^2^ analysis, no correlation was observed between tissue BMAG expression and age, sex, tumor size, tumor stages or histological types (Supplementary Table S1).

### 2.2. Expression of Tissue BMAG Was Associated With CCA Genesis in Hamster Model

**Figure 2:**
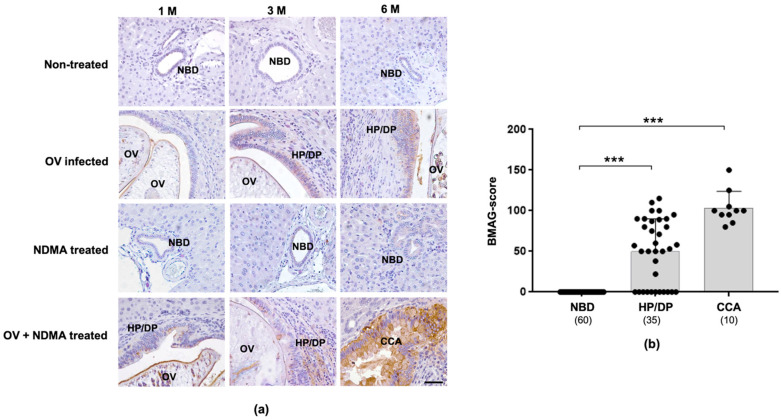
Expression of BMAG in CCA of hamsters (**a**) BMAG expression in the liver tissues from 4 groups of hamsters; (1) non-treated, (2) *Opisthorchis viverrini* (OV) infected, (3) NDMA treated and (4) CCA (OV + NDMA treated) groups, at 1, 3 and 6 months (M) after treatment. Scale-bar (—): 50 µm. (**b**) Bar-graph representing the mean values of BMAG-score in normal bile duct (NBD), hyperplastic/dysplastic bile duct (HP/DP) and CCA in hamster model. Stars (***) represent the statistically significant difference from NBD (*p* < 0.001). Black circle (•) represents BMAG-score of each individual case.

To investigate the association of BMAG expression and CCA genesis, BMA histochemistry was performed in hamster CCA tissues obtained from *Opisthorchis viverrini* (OV)-induced CCA hamsters. As shown in Figure 2a, BMAG was undetectable in normal bile ducts and hepatocytes in all treatment groups. The BMAG signals, however, were observed in pathologic (HP/DP) bile ducts, presented in the OV infected and OV + *N*-Nitrosodimethylamine (NDMA) treated groups as early as three months post-treatment, and the strong signal was observed in all CCA tissues developed in the OV + NDMA group at three and six months post-treatment. Hamster tissues in all groups were categorized based on the morphology and pathology of bile duct epithelia into: normal, HP/DP, and CCA. The expression levels of BMAG in these categorized tissues were compared, as shown in Figure 2b and the Supplementary Table S2. The expression of BMAG in CCA (103.5 ± 20.3) was significantly higher than those of HP/DP (50.3 ± 39.9) and normal bile ducts (*p* < 0.001), indicating the involvement of BMAG during CCA development.

### 2.3. BMAG Was Detected in the Patients’ Sera and Can Be a Diagnostic Marker for CCA

**Figure 3:**
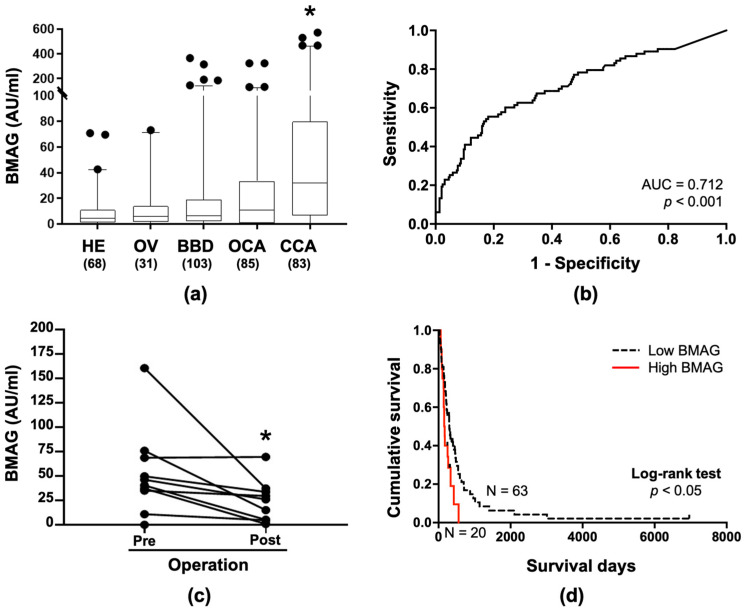
BMAG in the sera of CCA patients and non-CCA control. (**a**) Box-plot representing the serum BMAG levels determined by double BMA enzyme linked-lectin assay of healthy (HE), OV infected (OV), benign biliary diseases (BBD), other gastrointestinal cancers (OCA) and CCA. Black circle (•) represents BMAG level of each individual case. (**b**) ROC curve representing the power of serum BMAG level in distinguishing CCA patients from HE controls. AUC = area under curve (**c**) serum BMAG levels in corresponding pre- and post-operative sera (*n* = 10). (**d**) Survival analysis of CCA patients using log-rank test and Kaplan–Meier plot. Star (*) represents the statistically significant difference (*p* < 0.05). Number of cases is in the parentheses.

The positive signal of BMAG was observed in the luminal surface of CCA tissues from both patients (Figure 1a, inlet), and hamsters (Figure 2a), suggesting a secretory nature of BMAG glycoconjugate that may be detected in the sera. To explore this possibility, a double BMA-enzyme linked-lectin assay was established and determined BMAG levels in the sera from 83 CCA patients and 287 non-CCA control subjects, including 68 healthy control subjects, 31 OV infected (OV), 103 benign biliary diseases (BBD), and 85 other gastrointestinal cancers (OCA). The serum BMAG level of CCA patients was significantly higher than those of each control group (*p* < 0.01, Table 1 and Figure 3a). Analysis of serum BMAG among cancer cases revealed that the average level of serum BMAG from CCA patients was higher than those of other cancer groups tested but showed statistical significance only with those with colon cancer (Supplementary Figure S1).

Receiver operating characteristic (ROC) analysis revealed that BMAG can be a potential serum marker which significantly distinguished CCA patients from the controls with the area under curve of 0.712 (*p* < 0.001, Figure 3b). The cut-off value of serum BMAG at 26.6 AU/mL was suggested by the Youden index, providing 55.4% sensitivity, 81.9% specificity, 46.9% positive predictive value (PPV), 86.9% negative predictive value (NPV), 18.1% false positive, 44.6% false negative and 76.0% accuracy for the differentiation of CCA from non-CCA controls. In addition, BMAG levels in 10 pairs of corresponding pre- and post-operative sera were determined. The result showed that the BMAG level was significantly reduced after tumor removal (Figure 3c, *p* < 0.05, paired *t*-test), indicating the association of serum BMAG with tumor origin.

As serum CA19-9 is a standard marker for CCA, the diagnostic power of serum BMAG with serum CA19-9 were determined in the matched cases of 42 CCA and 231 non-CCA controls. As shown in Supplementary Table S3, serum BMAG provided a comparable diagnostic value with those of serum CA19-9.

### 2.4. Serum BMAG Was an Independent Poor Prognostic Indicator for CCA

To analyze the correlation between serum BMAG and the clinical data of patients, CCA patients were categorized into two groups according to the mean of serum BMAG; a low serum BMAG group (<82.5 AU/mL) and a high serum BMAG group (≥82.5 AU/mL). The univariate analysis indicated an association of serum BMAG with the sex of CCA patients (*p* < 0.01; Table 2). No correlation of serum BMAG with age and tumor size of patients was observed using Pearson correlation (Supplementary Table S4). The overall survival of CCA patients was 252 days (95% CI = 196.7−334.3). Kaplan–Meier analysis with a log-rank test revealed that CCA patients with high serum BMAG (N = 20) exhibited a significantly shorter survival period than those with low serum BMAG (N = 63) (*p* = 0.011, Figure 3c). The median survival times of the patients with high and low serum BMAG were 117 days (95% CI = 117.5–196.4) and 221 days (95% CI = 221.5–372.5), respectively. High serum BMAG with a hazard ratio (HR) of 1.960 and the tumor stage IVB with a HR of 2.198 were indicated by the Cox regression model for univariate analysis (Table 3). Using multivariate Cox regression analysis, serum BMAG was found to be an independent poor prognostic indicator for CCA with HR of 1.873, regardless of the tumor stage of the CCA (Table 3).

## 3. Discussion

Several lectin-based approaches have been developed for detection of CCA-associated glycans in tissues and/or sera of patients [2,5,6,9,10]. In this study, BMA, a lectin from *Butea monosperma* seeds, was used to detect the GalNAc/Gal glycans in clinical specimens from CCA patients, and the potential usage of BMA to discriminate CCA patients from non-CCA subjects was investigated. Using lectin-histochemistry, BMA binding glycans (BMAG) were detected in pre-neoplastic (HP/DP) bile ducts and CCA tissues from patients, but not in the normal bile ducts. The significance of BMAG in CCA development was demonstrated by the OV-induced CCA hamster model. High levels of BMAG could be detected in the sera of CCA patients and distinguished from those of non-CCA controls. Moreover, a high serum BMAG level was associated with a shorter survival time of CCA patients. These findings indicate a potential serum glycobiomarker of BMAG for diagnosis and prognosis of CCA.

Over the past decade, plant lectins have been used as tools for searching new glycobiomarkers for CCA [2,13]. *Butea monosperma* is a medicinal plant that exhibits many pharmacological effects, such as anti-microbial, anti-helminthic, anti-diabetic effects etc. [14]. In this study, BMA, a GalNAc/Gal binding lectin from the seeds of *Butea monosperma* [11], was used to detect BMAG in the tumor tissues and sera from CCA patients, and evaluate for the potential of being a glycobiomarker for diagnosis and prognosis of CCA. In contrast with hepatoma, the majority of patient CCA tissues tested exhibited BMAG signal with differential BMAG scores. The BMAG scores, however, were not associated with clinicopathological findings of CCA patients in this setting. As the BMAG signal was positive in almost all CCA cases but not hepatoma, lectin histochemistry of BMAG may be used to differentiate CCA from hepatoma.

A double BMA-enzyme linked-lectin assay was successfully developed to detect BMAG in patients’ sera. The levels of serum BMAG from CCA patients were significantly higher than those of the non-CCA groups and could differentiate CCA patients from the control groups with a comparable diagnostic power with the previous reported glycobiomarkers, such as CA19-9, SJA-binding glycan, and *Wisteria floribunda* agglutinin-positive mucin-1 [15]. A sensitivity of 63–68% and specificity of 73–88% was gained by these individual glycan markers. It is well known that the diagnostic power of individual test can be elevated when using a combination of two to three markers [15]. Combining serum BMAG with other CCA markers should be further investigated to improve the diagnostic power of CCA. In addition, high serum BMAG and tumor stage IVB were independently associated with the short survival of CCA patients in this cohort.

The expression levels of BMAG in bile ducts with HP/DP and CCA were not significantly different. BMAG levels in the serum of CCA patients, however, were significantly higher than those of BBD cases and hence serum BMAG could be used to distinguish between benign and malignant conditions. There are two possible explanations for this discrepancy. First, the number of BMAG producing cells in tumor (CCA cells) may be higher than those in HP/DP cells, resulting in the higher level of BMAG in the serum of CCA patients than those of BBD patients. Second, BMAG-carrier proteins expressed in CCA might be different from those expressed in hyperplasia/dysplasia. Identification of carrier proteins in hyperplasia/dysplasia and CCA is needed to address this point.

Alteration of glycosylation has been shown to be involved in tumor growth, metastasis and therapeutic resistance [16,17]. Several specific glycans are applicable as markers for diagnosis, monitoring and prognostic prediction of many cancer types [18,19]. In CCA, several CCA-associated glycans are involved in tumor progression and are suggested to be the targets for CCA treatment [19]. Indramanee et al. used histochemistry of 14 commercially available lectins to detect alteration of glycans in patient CCA tissues, and found that almost all lectins tested gave positive signals with normal bile duct, HP/DP and CCA, including hepatocytes and stromal cells [3]. SJA was the only GalNAc/Gal binding lectin that bound to HP/DP and CCA but not normal bile duct and stromal cells. Similar to BMA, SJA binding Gal/GalNAc glycan (SNAG) could also be detected in the patients’ sera with a lower sensitivity (59.5%) and specificity (73.6%) [9]. BMA recognizes glycan structures similar but not identical to SJA. SJA has a specificity toward T antigens (Gal-β-1,3-GalNAc) [20,21], whereas BMA had the highest affinity to GalNAc [11].

As shown in Figure 3c, a high serum BMAG level was associated with poor prognosis of CCA, implying an association of BMAG with CCA progression. The functional analyses in CCA cell lines, namely KKU-213, KKU213-L5, KKU-214, and KKU-214L5, showed that BMA significantly reduced the migration and invasion ability of CCA cell lines (*p* < 0.05) [22]. The mechanism underlying the involvement of BMAG in CCA metastasis should be further investigated.

In conclusion, this present study demonstrated the elevation of a novel CCA-associated glycan, BMAG, in the pre-neoplastic (HP/DP) bile ducts and CCA. BMAG was demonstrated to be a potential glycobiomarker for diagnosis and prognosis of CCA. Further studies on the mechanism and significance of BMAG in CCA development may provide a better understanding of glycobiology in cancer, which could possibly lead to the curative treatment of CCA.

## 4. Materials and Methods

### 4.1. Patients’ Tissues and Sera

Paraffin-embedded tissues and serum samples were obtained from the specimen bank of the Cholangiocarcinoma Research Institute, Khon Kaen University, Thailand. Informed consent was obtained from each subject and the research protocol was approved by the ethics committee for human research of Khon Kaen University (HE611318).

Paraffin-embedded liver sections from patients with 74 CCA, 10 BBD and 8 hepatomas were included in this study. Pre-operative serum samples were collected from the histologically proven cases of 83 CCA, 103 BBD (12 biliary adenoma, 4 biliary cyst/papillomatosis, 12 cholecystitis/cholangitis, 2 cirrhosis, 49 periductal fibrosis, 1 hemangioma, 2 gall stone, 9 biliary inflammation, and 12 others), and 85 OCA (14 ampulla of vater, 15 colorectal, 5 gall bladder, 29 hepatoma, 13 stomach, and 9 pancreas). Post-operative sera obtained at least 3 months after surgical treatment from 10 CCA patients were collected. The OV infected subjects (N = 31) were identified by fecal OV egg examination. Sera of asymptomatic healthy control subjects (N = 68), defined by normal fasting blood glucose and liver functions (aspartate amino transferase, AST; alanine amino transferase, ALT; alkaline phosphatase, ALP), were collected during the annual check-up at Srinagarind Hospital, Faculty of Medicine, Khon Kaen University. Serum CA19-9 was obtained from the records of the routine analysis of Srinagarind Hospital (Roche Diagnostics GmbH) according to the International Federation of Clinical Chemistry and Laboratory Medicine (IFCC).

### 4.2. CCA Hamster Tissues

Paraffin-embedded hamster liver sections were obtained from the previous study [23], under an approval by the Animal Ethics Committee of Khon Kaen University (AEMDKKU1/2558). Samples were collected from 4 groups of experimental hamsters: non-treated control, OV infected, NDMA treated and OV + NDMA treated groups, as previously described [24]. BMAG expression was examined in 5 representative tissue sections per group at 1, 3 and 6 months after the beginning of experiments.

### 4.3. Purification and Biotinylation of Butea monosperma Agglutinin

BMA was purified from *Butea monosperma* seeds using 50–80% ammonium sulfate precipitation and GalNAc-agarose affinity chromatography, as described previously [11]. Biotinylated BMA was prepared using an EZ-Link Sulfo-NHS-LC-Biotin kit (Pierce/Thermo Fisher Scientific, Rockford, IL, USA) according to the manufacturer’s recommendation.

### 4.4. BMA Histochemistry

The expression of BMAG in CCA tissues from CCA patients and experimental CCA in the hamster liver was determined using lectin histochemistry [3]. After deparaffinization and blockage of non-specific reactivity using 0.5% BSA in phosphate buffer saline (PBS), the sections were incubated with 3 µg/mL of biotinylated BMA at room temperature overnight, followed by 1:100 dilution of horseradish peroxidase (HRP)-conjugated streptavidin (streptavidin-HRP, Invitrogen, Camarillo, CA, USA), at room temperature for 40 min. The staining signal was developed using 25 mg/mL of diaminobenzidine tetrahydrochloride (Sigma Aldrich, St. Louis, MO, USA) and counterstained with Mayer’s hematoxylin (Bio-Optica, Milano, Italy). Slides incubated with PBS instead of biotinylated BMA or streptavidin-HRP were used as negative controls of the detection system. The specificity of the lectin was shown by the negative signal in the slide incubated in the presence of 200 mM GalNAc (Supplementary Figure S2). The expression level of BMAG was presented as a BMAG-score (0–300), calculated from the multiplication of staining intensity (negative = 0, weak = 1, intermediate = 2, strong = 3), with a percentage of positive cells (0–100%) [25].

### 4.5. Double BMA Sandwich Enzyme-Linked Lectin Assay

The level of serum BMAG was determined using double BMA sandwich enzyme-linked lectin assay. Each well of a 96 well-plate was coated with 50 µL of 0.25 µg/mL of BMA at 4 °C overnight. After washing with 0.1% Tween-20 in PBS pH 7.4 (PBST) and blocking non-specific binding with 3% BSA in PBST at room temperature for 1 h, the plate was incubated with 50 µL of 1:10 serum sample in 1% BSA-PBST at room temperature for 1 h. After incubation, the sample was removed, and the plate was washed 5 times with PBST. BMAG was detected by incubating with 0.5 µg/mL of biotinylated-BMA at room temperature for 1 h, followed by 1:10,000 diluted streptavidin-HRP (Invitrogen, Camarillo, CA, USA) at room temperature for 1 h. After 5 times washing, the signal was developed by incubating with tetramethylbenzidine (TMB)-substrate (Sigma Aldrich, St. Louis, MO, USA) in the dark at room temperature for 15 min. The reaction was stopped using 50 µL of 1 N H_2_SO_4_ and the absorbance was measured at 450 nm. The serum BMAG level was calculated and presented as arbitrary units (AU)/mL based on the standard control prepared from cell lysate of KKU-213A, a human CCA cell line [26]. The level of BMAG in each sample was an average value from duplicate measurements.

### 4.6. Statistical Analysis

Statistical analyses were performed using SPSS 17.0 software (SPSS, Chicago, IL, USA) and graphs were generated using GraphPad Prism 8 (GraphPad Inc., La Jolla, CA, USA). Expression levels of BMAG in CCA tissues and sera were presented as the mean with standard deviations (SD). The differences of BMAG levels between groups were compared using Student’s *t*-test. Correlation between BMAG levels and clinicopathological data of CCA patients was analyzed using a Chi-square (χ^2^) test. The correlation between BMAG levels and continuous variables, age and tumor size, were analyzed using a Pearson correlation. Survival analysis was performed using a Kaplan–Meier plot and log-rank test. A Cox proportional-hazards regression was used to analyze the hazard ratio and multivariate survival analysis. The variables with *p* < 0.1 in the Univariate Cox-Regression analysis were selected for a Multivariate Cox-Regression analysis. *p* < 0.05 was considered as statistical significance.

## Figures and Tables

**Table 1:** Level of serum-BMAG in CCA patients and non-CCA controls.

	Subjects				
	HE	OV	BBD	OCA	CCA
**Number of cases**	68	31	103	85	83
**Level of BMAG** (AU/mL)					
Mean ± SD	9.4 ± 14.7	11.2 ± 17.5	25.0 ± 56.5	27.8 ± 54.2	82.5 ± 128.9
***p*-value (*t*-Test)**					
vs HE	-	0.612	0.027	0.007	<0.001
vs OV	-	-	0.182	0.096	0.002
vs BBD	-	-	-	0.730	<0.001
vs OCA	-	-	-	-	<0.001

AU = Arbitrary Unit; HE = healthy controls; OV = *Opisthorchis viverrini* infected patients, BBD = benign biliary diseases, OCA = other gastrointestinal cancers, CCA = cholangiocarcinoma, SD = standard deviation.

**Table 2:** Correlation of serum-BMAG and clinicopathological data of CCA patients.

Variables	N	Serum-BMAG		*p*-Value
		<82.5 AU/mL	≥82.5 AU/mL	
**Sex**				0.009
Female	26	15	11	
Male	57	48	9	
**Age**				0.920
<56 years old	34	27	7	
≥56 years old	49	36	13	
**Tumor size**				0.515
<7 cm	45	35	10	
≥7 cm	35	25	10	
**Histological types**				0.793
Papillary type	23	17	6	
Non-papillary type	60	46	14	
**Tumor stages**				0.855
I–III	16	13	3	
IVA	39	29	10	
IVB	28	21	7	

AU/mL = arbitrary unit per milliliter; cm = centimeter.

**Table 3:** Cox regression analysis of serum-BMAG in CCA patients.

Variables	Univariate Analysis			Multivariate Analysis		
	HR	95% CI	*p*-Value	HR	95% CI	*p*-Value
**Sex**				-	-	-
Female	1					
Male	0.978	0.604–1.581	0.926			
**Age**				-	-	-
<56 years old	1					
≥56 years old	1.266	0.800–2.004	0.314			
**Tumor size**				-	-	-
<7 cm	1					
≥7 cm	1.312	0.837–2.055	0.237			
**Histological types**				-	-	-
Papillary type	1					
Non-papillary type	1.353	0.834–2.212	0.219			
**Tumor stages**						
I–III	1			1		
IVA	1.295	0.709–2.367	0.391	1.224	0.667–2.245	0.533
IVB	2.198	1.157–4.174	0.016	2.068	1.084–3.945	0.009
**Serum-BMAG**						
BMAG < 82.5 AU/mL	1			1		
BMAG ≥ 82.5 AU/mL	1.960	1.151–3.336	0.013	1.873	1.096–3.199	0.022

AU/mL = arbitrary unit per milliliter; CI = confidence interval; cm = centimeter; HR = hazard ratio.

## Supplementary Materials

The following are available online, Figure S1: BMAG expression in CCA compared with other cancers, Figure S2: BMAG lectin histochemistry optimization, Table S1: Tissue BMAG-score and clinicopathological data correlation, Table S2: BMAG expression in bile duct epithelia of liver tissues from hamster model, Table S3: Diagnostic values of serum BMAG and serum CA19-9, Table S4: Pearson correlation of BMAG with age and tumor size of CCA patients.

## Sample Availability

Samples of the compounds are not available from the authors.
