# Supplementary material for: A Novel Serum Glycobiomarker for Diagnosis and Prognosis of Cholangiocarcinoma Detected by Butea monosperma Agglutinin
Source: Molecules. 2021 May 8;26(9):2782. doi: 10.3390/molecules26092782 (PMC8125881; doi:10.3390/molecules26092782)
Supplement: Supplementary file 1 [file molecules-26-02782-s001.zip › molecules-1190718-supplementary.pdf]

Teeravirote_Supplementary Data

Supplementary Table S1 Tissue BMAG-score and clinicopathological data correlation

Variables	No. of patients	Tissue-BMAG score		*P*-value
		Low (<107)	High (≥ 107)	
**Sex**				
Female	24	12	12	*0.598*
Male	50	25	25	
**Age**				
<56 years	37	16	21	*0.176*
≥56 years	37	21	16	
**Tumor size**				
<7 cm	31	17	14	*0.319*
≥7 cm	43	20	23	
**Histological types**				
Papillary type	24	14	10	*0.228*
Non-papillary type	50	23	27	
**Tumor stages**				
I-III	29	16	13	*0.124*
IVA	35	19	16	
IVB	10	2	8	

Supplementary Table S2 BMAG expression in bile duct epithelia of liver tissues from hamster model

Hamster groups	Code	BMAG score		
		NBD	HP/DP	CCA
**Non-treated**				
1 month	H1M01	0	ND	ND
	H1M02	0	ND	ND
	H1M03	0	ND	ND
	H1M04	0	ND	ND
	H1M05	0	ND	ND
3 months	H3M01	0	ND	ND
	H3M02	0	ND	ND
	H3M03	0	ND	ND
	H3M04	0	ND	ND
	H3M05	0	ND	ND
6 months	H6M01	0	ND	ND
	H6M02	0	ND	ND
	H6M03	0	ND	ND
	H6M04	0	ND	ND
	H6M05	0	ND	ND
**OV infected**				
1 month	HV1M01	0	50	ND
	HV1M02	0	38	ND
	HV1M03	0	0	ND
	HV1M04	0	58	ND
	HV1M05	0	0	ND
3 months	HV3M01	0	0	ND
	HV3M02	0	50	ND

Hamster groups	Code	BMAG score		
		NBD	HP/DP	CCA
	HV3M03	0	53	ND
	HV3M04	0	89	ND
	HV3M05	0	0	ND
6 months	HV6M01	0	80	ND
	HV6M02	0	87	ND
	HV6M03	0	90	ND
	HV6M04	0	95	ND
	HV6M05	0	50	ND
**NDMA treated**				
1 month	HD1M01	0	ND	ND
	HD1M02	0	ND	ND
	HD1M03	0	ND	ND
	HD1M04	0	ND	ND
	HD1M05	0	ND	ND
3 months	HD3M01	0	ND	ND
	HD3M02	0	ND	ND
	HD3M03	0	ND	ND
	HD3M04	0	ND	ND
	HD3M05	0	ND	ND
6 months	HD6M01	0	100	ND
	HD6M02	0	0	ND
	HD6M03	0	0	ND
	HD6M04	0	0	ND
	HD6M05	0	0	ND
**OV + NDMA**				
1 month	HVD1M01	0	0	ND
	HVD1M02	0	57	ND
	HVD1M03	0	0	ND
	HVD1M04	0	0	ND
	HVD1M05	0	71	ND
3 months	HVD3M01	0	22	80
	HVD3M02	0	75	150
	HVD3M03	0	80	100
	HVD3M04	0	110	95
	HVD3M05	0	90	105
6 months	HVD6M01	0	50	125
	HVD6M02	0	60	95
	HVD6M03	0	115	100
	HVD6M04	0	90	85
	HVD6M05	0	100	100
**Mean**		**0.0**	**50.3**	**103.5**
**SD**		**0.0**	**39.9**	**20.3**

CCA = cholangiocarcinoma; HP/DP = dysplasia/hyperplasia; NBD = normal bile ducts; ND = Not detected; NDMA = *N*-Nitrosodimethylamine ; OV = *Opisthorchis viverrini*; SD = standard deviation

Supplementary Table S3 Diagnostic values of serum BMAG and serum CA19-9

Diagnostic values[#]	BMAG (cut off 26.6 AU/ml)	CA19-9 (cut off 37 U/ml)
% Sensitivity	42.9	71.4
% Specificity	90.9	83.1
% Positive predictive value	46.2	43.5
% Negative predictive value	89.7	94.1
% False positive	9.1	16.9
% False negative	57.1	28.6
% Accuracy	83.5	81.3

CA19-9 = carbohydrate 19-9; [#]This diagnostic values were calculated base on 42 CCA and 231 non-CCA cases.

Supplementary Table S4 Pearson correlation of BMAG with age and tumor size of CCA patients

Pearson correlation	BMAG *vs* age	BMAG *vs* tumor size
r	0.173	-0.083
P-values	0.118	0.460

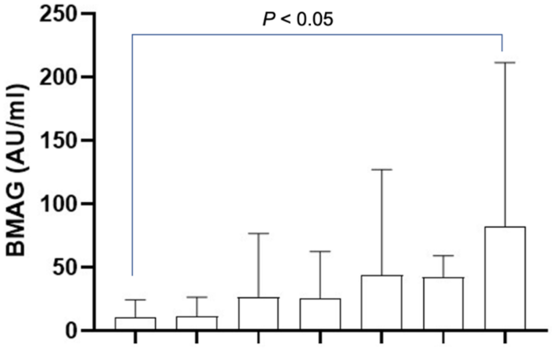

Types of cancer	CC	GC	PC	AM	HCC	GB	CCA
Number of case	15	13	9	14	29	5	93
Serum BMAG level (AU/ml)							
mean	10.84	11.52	26.74	25.93	43.98	42.37	82.51
SD	13.54	14.99	49.88	36.53	83.08	16.67	128.92
P-vaue (T-test, *vs* CCA)	***0.034***	*0.051*	*0.203*	*0.107*	*0.142*	*0.490*	-

Supplementary Figure S1 BMAG expression in CCA compared with colon (CC), gastric (GC), pancreas 9PC), ampular of vater (AMC), hepatoma (HCC) and gall bladder (GB) cancers.

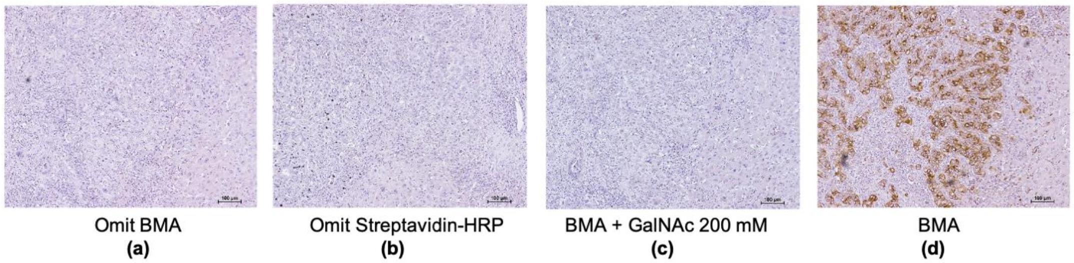

Supplementary Figure S2. BMAG lectin histochemistry optimization, the negative controls are (a) using PBS instead of lectin (omit lectin), (b) omit streptavidin-HRP, and (c) neutralizing BMA by 200 mM N-acetuyl galactosamine (GalNAc). (d) BMA was used for lectin histochemistry of CCA tissues.
